# Early interactions with newly diagnosed TB patients in hospital can support linkage to care

**DOI:** 10.5588/pha.22.0012

**Published:** 2022-09-21

**Authors:** L. Viljoen, P. Hendricks, G. Hoddinott, N. Vanqa, M. Osman, A. C. Hesseling, S-A. Meehan

**Affiliations:** 1 Desmond Tutu TB Centre, Department of Paediatrics and Child Health, Faculty of Medicine and Health Sciences, Stellenbosch University, South Africa; 2 School of Human Sciences, Faculty of Education, Health and Human Sciences, University of Greenwich, Greenwich, UK

**Keywords:** tuberculosis, linkage to care, patient engagement, TB cascade

## Abstract

**BACKGROUND:**

In South Africa, failure to link individuals diagnosed with TB to care remains an important gap in the TB care cascade. Compared to people diagnosed at primary healthcare (PHC) facilities, people diagnosed in hospitals are more likely to require additional support to be linked with PHC TB treatment services. We describe a patient interaction process to support linkage to TB care.

**METHODS:**

We implemented a step-by-step early patient interaction process with 84 adults newly diagnosed with TB in one district hospital in Khayelitsha, Cape Town, South Africa (August 2020–March 2021). We confirmed patient contact details, provided TB and health information, shared information on accessing care at PHC facilities and answered patients’ questions in their home language.

**RESULTS:**

Most patients (54/84, 64%) provided updated telephone numbers, and 19/84 (23%) reported changes in their physical address. Patients welcomed practical and health information in their home language. The majority (74/84, 88%) were linked to care after hospital discharge.

**CONCLUSIONS:**

A simple early patient interaction process implemented as part of routine care is a feasible strategy to facilitate early TB treatment initiation and registration.

## Abstract (fr)

**CONTEXTE:**

En Afrique du Sud, l’incapacité à relier les personnes dont la TB a été diagnostiquée aux soins reste une lacune importante dans la cascade des soins antituberculeux. Comparativement aux personnes diagnostiquées dans les établissements de soins de santé primaires (PHC), les personnes diagnostiquées dans les hôpitaux sont plus susceptibles d’avoir besoin d’un soutien supplémentaire pour être reliées aux services de traitement de la TB des PHC. Nous décrivons un processus d’interaction avec le patient pour favoriser le lien avec les soins antituberculeux.

**MÉTHODES:**

Nous avons mis en œuvre un processus d’interaction précoce, étape par étape, avec 84 adultes chez qui la TB a été récemment diagnostiquée dans un hôpital de district de Khayelitsha, au Cap, en Afrique du Sud (août 2020–mars 2021). Nous avons confirmé les coordonnées des patients, fourni des informations sur la TB et la santé, partagé des informations sur l’accès aux soins dans les établissements de PHC et répondu aux questions des patients dans leur langue maternelle.

**RÉSULTATS:**

La plupart des patients (54/84 ; 64%) ont fourni des numéros de téléphone actualisés, et 19/84 (23%) ont signalé des changements dans leur adresse physique. Les patients ont apprécié les informations pratiques et ceux ayant trait à la santé dans leur langue maternelle. La majorité d’entre eux (74/84 ; 88%) ont été reliés aux soins après leur sortie de l’hôpital.

**CONCLUSIONS:**

Un processus simple d’interaction précoce avec le patient, mis en œuvre dans le cadre des soins de routine, est une stratégie réalisable pour faciliter l’initiation et l’enregistrement précoce du traitement de la TB.

TB is a global health crisis, with nearly 10 million people estimated to have developed the disease in 2020.^1^ South Africa is one of eight countries accounting for two thirds of global TB cases.^1^ The 2018 National TB Prevalence Survey found an incidence of ~677/100,000 population—equivalent to 390,000 cases per year.^2^ The TB care cascade is a useful tool to identify gaps in the TB programme.^3^ Using previously published methods,^4^ an updated South African TB care cascade for 2018 showed that of those estimated to have TB, 80% were diagnosed, 60% were notified and registered as having started treatment and 48% completed treatment successfully. A key gap in the South African TB case cascade is that diagnosed patients are not reliably included in the national TB treatment register and not successfully linked to care.^5^–^7^ To note, patients diagnosed in hospital are less likely to be linked to care than patients diagnosed at primary healthcare (PHC) facilities.^8^ Adults with TB not receiving treatment are at substantial risk of morbidity and mortality, and may contribute to ongoing TB transmission.^5^,^8^–^10^

In South Africa, the majority of people are tested for TB at public facilities (community-based PHC clinics or hospitals), community-based outreach programmes or mobile testing sites.^11^–^13^ Patients diagnosed at the hospital level are referred to PHC facilities for registration in local treatment registers to receive TB care or to a TB specialised hospital if inpatient care is required. In the TB healthcare system in Cape Town, people diagnosed with TB are routinely recorded in a treatment register linked across facilities, which allows for treatment initiation at any PHC. Patients are provided with a referral letter and are typically required to attend (in person) a local PHC where they are registered, assessed and receive treatment (i.e., linkage). Patients who are not linked to a TB treatment facility are visited at home at the addresses provided in their patient files by community health workers (CHWs). Patients who are lost to follow-up after diagnosis but before treatment initiation have been described as being “pre-treatment lost to follow-up”.^14^ We used ‘initial loss to follow-up’ (ILTFU) to describe patients who were newly diagnosed with TB (at hospital level) but were not linked to care at a PHC facility within 30 days of discharge, regardless of whether initial treatment was dispensed in hospital.

A previous systematic review showed that the health system barriers influencing TB treatment initiation in sub-Saharan Africa include health providers’ lack of treatment knowledge, negative health worker attitudes and information management challenges.^15^ Patient-level factors include lengthy diagnostic and treatment delays and patients dying before they were able to initiate care.^15^ Negative perceptions, lack of knowledge of public healthcare and social factors and economic burdens, including family and school responsibility, have also been reported as treatment initiation barriers.^15^,^16^ TB patients have reported that receiving limited information about their TB diagnosis, lack of instruction on how and where to link to care, negative experiences related to previous TB episodes and having alternative explanations for their TB symptoms contributed to delays in linkage to treatment.^17^ Researchers recently found that a dedicated TB referral service in a paediatric hospital setting in South Africa can reduce ILTFU and improve childhood TB reporting and registration.^7^

We drew on patient engagement—defined as supporting patients to “choose to participate in care in a way uniquely appropriate to the individual, in cooperation with a healthcare provider or institution, for the purpose of maximising outcomes”^18^—to inform a process of interacting with patients as part of routine care to improve linkage for hospital-diagnosed TB patients. These patients tend to have more severe TB and higher TB mortality than patients diagnosed at community level.^8^,^17^,^19^ We interacted with TB patients (or their caregivers) routinely diagnosed with TB at a district-level hospital in South Africa prior to discharge to facilitate linkage to a PHC facility. This study describes 1) the processes used to interact with newly diagnosed TB patients, 2) the demographic, clinical and linkage profile of patients, and 3) the feasibility and effort required to support this approach. This was a pragmatic study design nested in routine support for patient linkage to care. In this article, we present our lessons learnt from the patient interaction process.

## METHODS

### Setting

This study was nested in a larger study, the LINKEDin Study. The Khayelitsha health sub-district in Cape Town, Western Cape Province, was one area where the LINKEDin Study was implemented. Khayelitsha is peri-urban and serviced by a district hospital, the Khayelitsha District Hospital (KDH), and 13 PHC facilities providing TB services. Khayelitsha has a high burden of TB and HIV, with ~4,310 TB patients registered in the drug-susceptible TB treatment register in 2019, and 31.3% of pregnant women reportedly being HIV-positive in 2017.^20^–^22^

KDH is a 340-bed hospital, which offers a 24-hour Emergency Centre, an outpatient department and medical, surgical, obstetrics, gynaecology and paediatric wards. On admission, patients at KDH are registered on an electronic patient management system providing a unique patient identifier. KDH does not register and notify TB patients. TB patients are only registered when they access a PHC facility or are transferred for further inpatient care to a TB treatment hospital and entered into the TB treatment register at these facilities.

### Study design

This was a pragmatic feasibility study where we describe the process of implementing a step-by-step patient interaction process to facilitate linkage to TB care after hospital discharge. This work is part of the LINKEDin Study that aimed to reduce ILTFU among TB patients at public facilities in three high TB burden provinces of South Africa (Gauteng, KwaZulu-Natal and Western Cape). LINKEDin included a combination of interventions, including text message reminders, phone calls and household contact tracing to improve linkage to care. Findings from LINKEDin (2018–2020) showed that TB patients diagnosed in hospital were less likely to be linked to a PHC facility for treatment (ILTFU: 43.7%) than those diagnosed at PHC facilities (ILTFU: 11.8%).^19^ TB patients diagnosed in hospital reported that they did not receive adequate information at discharge, did not know that they needed to attend a local PHC facility for further treatment or were unaware of their TB diagnosis.^17^ LINKEDin Study staff reported that many patients had incorrect or inadequate contact details, making it difficult to trace and refer them for further care. To address these issues, we implemented an interactive linkage to PHC facility care process for TB patients diagnosed in the hospital. This was not part of the specified LINKEDin intervention (described above), but rather steps implemented to support patient linkage at one hospital within the LINKEDin Study.

### Sampling

Between 31 August 2020 and 15 January 2021, we included all newly diagnosed TB patients admitted at KDH on a daily basis. Patients diagnosed in outpatient departments were not admitted and were not present to be included in the simple hospital-based interaction process. The list of newly diagnosed patients was generated by the Western Cape Provincial Health Data Centre (PHDC), which collects and integrates information from all available individual-level electronic health records from public healthcare providers.^23^

### Data collection and analysis

Study data were collected and managed using Research Electronic Data Capture (REDCap; Vanderbilt University, Nashville, TN, USA) tools hosted at Stellenbosch University.^24^,^25^ REDCap is a secure, web-based software platform designed to support data capture for research studies. We documented whether we were able to physically locate the patient and interact with them, and subsequently searched the PDHC to confirm linkage to TB care after discharge.

The interaction process was implemented by two trained LINKEDin staff members who were part of the study and familiar with the PHDC and TB care processes in the local setting. The ~20-minute interactions were presented in conjunction with the routine care of patients in consultation with their healthcare providers and were conducted in the patients’ language of choice (Xhosa, Afrikaans or English).

Patient interactions were designed to achieve three key goals: 1) confirming patient personal information and contact details, 2) providing patients with logistical, general and TB health information, including information on linkage to TB services; and 3) answering patient questions.

**FIGURE 1.**
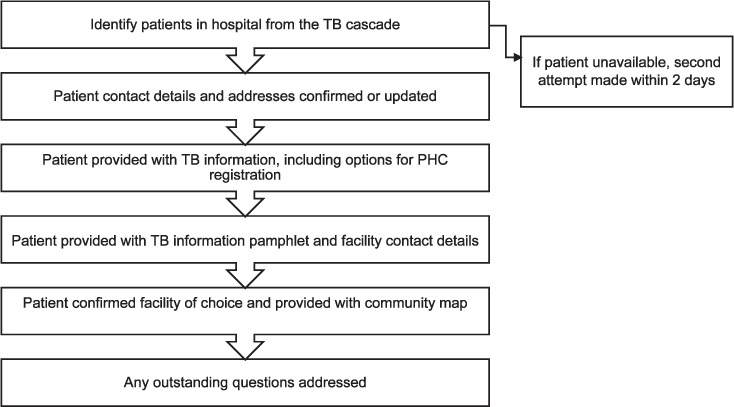
The patient interaction process. PHC = primary healthcare.

The patient interaction process followed six pre-determined steps (Figure 1). Physically locating all patients diagnosed at KDH using the TB cascade included daily confirmation of the names and wards of newly diagnosed TB patients. At least two attempts were made to locate each patient in-person before hospital discharge. Patient interactions were scheduled directly after routine clinical rounds to reduce the likelihood of missing patients discharged or transferred to other hospitals that morning.

Patients were asked to confirm their contact details on file, including physical address (and proximate landmarks for informal addresses) and contact number(s). Next, patients were informed of options for their ongoing TB treatment and registration. We provided printed materials that patients could keep, including a printed map and contact details for the preferred PHC facilities where they might be linked to care.

**FIGURE 2.**
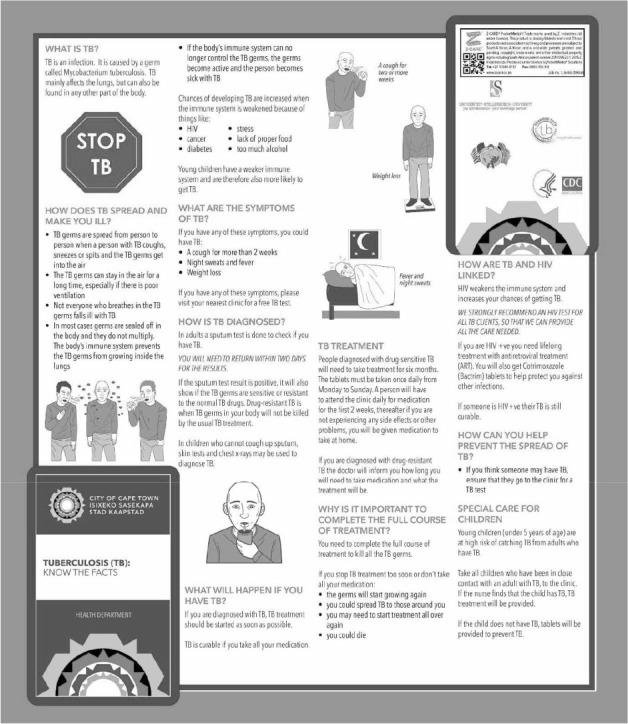
Example of pamphlets distributed to TB patients. Source: City of Cape Town Health Department, Cape Town, South Africa.

Patients were then given TB information pamphlets in their home language, provided guidance on the TB treatment process, the importance of continuing their TB treatment and information on TB prevention for their contacts (Figure 2). Finally, patients were given the opportunity to ask questions.

After interactions, we captured patient demographic, clinical and TB linkage data from the PHDC into the REDCap database. Time to linkage was recorded as the number of days from hospital discharge to linkage to care and registration at a PHC facility.

We analysed the data descriptively to characterise the patient profile, and described and reflected on the lessons learnt from our interaction process. Reflection is described as a “tool for shaping thoughts, ideas, and beliefs … [which] enables us to evaluate experience, learn from mistakes, repeat successes, revise, and plan”.^26^ Our reflections were conducted in biweekly virtual meetings over a period of 6 months with members of the study team. This iterative process was conducted after a draft report about the findings had been reviewed by stakeholders.

### Ethical considerations

The study was approved by the Health Research Ethics Committee of Stellenbosch University, Tygerberg (N18/07/069), the Western Cape Department of Health, Cape Town (NHRD ref: WC_201808_034) and the City of Cape Town Health Directorate, Cape Town, South Africa (study ID number 8053). We had a waiver of informed consent to access routine health data for TB patients.

## RESULTS

A total of 169 patients were identified in the PHDC as newly diagnosed with TB and were still admitted at KDH between 31 August 2020 and 15 January 2021. Of these, we could physically locate and interact with 84/169 (50%) prior to their discharge. Patients were predominantly male (57%), and 44 (58%) were between the ages of 25 and 44.

After two attempts had been made, we were unable to interact with 85/169 (50%) patients. Of these, 28 had been transferred to specialised TB hospitals (considered linked to care), 45 had already been discharged, 6 could not be located but were likely to have been discharged and 6 had died in hospital.

### Changes in contact details

Most patients provided updated contact details compared to what was captured in the routine electronic or paper records. Approximately two thirds of patients (54/84, 64%) provided updated telephone numbers and 19/84 (23%) patients indicated a change in residency or physical address. Several patients lived in informal areas where street addresses are difficult to determine and we made a note of directions, including relevant physical landmarks, to locate the home in patient folders.

### Linking of patients to care

Most patients contacted (74/84, 88%) were linked to a TB treatment facility after discharge. Of these, 47/74 (64%) were linked within 7 days of discharge, while 24/74 (32%) were linked between 8 and 30 days. Three remaining patients (4%) were linked to care after 30 days. While we did not elicit specific reasons for the variable delay in seeking care at the PHC, we have previously found that delayed linkage to treatment has been linked to patients having to come to terms with their diagnosis, and the prospect of interacting with the public health system after previous unpleasant TB treatment episodes.^17^ Of the 10 patients not linked, most had died (6/10, 60%) following discharge from hospital prior to linkage. There were no differences in demographic, disease or hospital admission profile between people who were and those who were not linked to care (Table).

**TABLE:** Profile of TB patients linked to care compared to those not linked to care after receiving the step-by-step patient interaction while in hospital

	Linked to TB care (*n* = 74)		Not linked to TB care (*n* = 10)	
	
	*n*	%	*n*	%
Age, years				
<15	2	3	0	0
15–24	5	7	0	0
25–34	22	30	2	20
35–44	21	28	3	30
45–54	11	15	2	20
55–64	8	11	2	20
>64	5	6	1	10
Sex				
Male	43	58	4	40
Female	31	42	6	60
Vitality status post discharge until the point of linkage				
Alive	74	100	4	40
Deceased	0	0	6	60
Linkage to TB treatment (days from discharge date )				
0–7	47	63	0	0
8–14	14	19	0	0
15–30	10	14	0	0
>30	3	4	0	0

### Reflections on feasibility and effort required for linkage

We concluded that ~20-minute targeted interactions with patients were feasible as it could be easily implemented by routine in-hospital staff with minimal effort. Given that half of the patients identified from the PHDC could not be located in hospital, more efficient strategies to reach patients prior to discharge are required. For example, early patient interactions could be done as part of the diagnosis or discharge process at the time that the patient leaves the ward.

Anecdotally, patients welcomed health information on their diagnosis, their local PHC and the prevention of TB transmission. Patients noted that they frequently had questions about TB and care in general and welcomed the distribution of pamphlets in their home language.

## DISCUSSION AND RECOMMENDATIONS

We found that 1) most patients had inadequate contact details on file, and engaging in the interaction process led to updated information, 2) patients appreciated the opportunity to learn more about their diagnosis and ask questions, and 3) the interactions provided patients with some autonomy to make decisions about their health, including where to access TB treatment. From our reflections, there are indications that an early patient interaction process can facilitate linkage to TB care for patients diagnosed in hospital. The implementation of the step-by-step process is a means to enable patients to better understand their diagnoses and allow patients to better manage their own health, which is in line with the patient centred care approach proposed in the WHO End TB strategy.^27^ From a health services perspective, collecting updated contact information meant that accurate information could be shared with PHC facilities that could be used to locate patients after discharge and support linkage to TB care.

A strength of this study was its pragmatic design nested in routine support for linking patients to care. This nesting in routine care means we included no formal process or outcome evaluation and no comparison group, limiting our ability to extrapolate from these findings—these are suggested lessons learnt, not proofs of effect. In this simple feasibility study, we did not account for other potential factors that might influence or confound linkage to care. For example, the COVID-19 pandemic might have delayed linkage to care and negatively impacted TB mortality. Patients may have been less motivated to provide their personal information during the pandemic for fear of disclosure. Health services had also been reorganised and TB resources reallocated to focus on the COVID-19 response. This may have negatively impacted the collection of accurate patient information in the absence of our additional interaction process. We also did not measure distance from home address to PHC facility. The availability of TB treatment services in this setting was good (12 PHC facilities providing TB treatment).

A strength of the study was the iterative process of internal reflection we used to develop our ‘lessons learnt’. The findings only draw from one hospital setting in one province of South Africa. While the hospital is in an area with the largest TB burden, findings are only transferable to similar hospital settings. However, more importantly, our main findings such as updating of contact details and providing practical information are good practices in general across all settings, i.e., not specific to hospital settings.

As a further limitation, the study team worked only on week-days and would have missed patients discharged over weekends. If the patient interaction process were included as part of routine care, fewer patients would be missed.

Delayed linkage to treatment offers substantial challenges to effective treatment implementation. Delayed linkage has been linked to patients having to come to terms with a diagnosis, facing the health system after previous unpleasant TB treatment episodes and practical considerations (taking time out from work, organising childcare) to access treatment. In South Africa, studies have implemented elements of patient engagement to reduce patient ILTFU through additional case support managers, various text message reminders and strengthening of ward-based out-reach teams.^28^–^30^ These patient engagement interventions involve resource-intensive processes focusing on the needs of patients. Our comparatively simple patient interaction process focusing on updating patient contact details, providing practical information and allowing patients the opportunity to ask questions was also aimed at improving the health outcomes of patients.

Interactive patient engagement processes outside of the TB programme interventions have improved patient treatment adherence and linkage to care.^31^ Researchers have found that effective elements of patient engagement vary by study, health condition and location.^32^,^33^ In other contexts, researchers found that being able to ask health providers questions is the foremost priority among patients in engagement interventions.^33^ These results are supported by interactions with patients in our study.

Although there are multiple benefits to implementing detailed and substantive patient engagement interventions, these programmes can be labour-intensive and time-consuming.^34^ We show that adapting engagement principles for a simple interaction with TB patients in hospital is feasible. Embedding this process within the patient discharge process using staff already involved during discharge would make this process cost and time-efficient. However, it would require updated and availability of TB diagnosis data.

This short interaction intervention ensured that patients had accurate information about their TB diagnosis, that they were empowered to access care after discharge and that the health system would have updated patient contact details. Community health workers and other public health initiatives, such as contact tracers, are able to locate patients who need support. While we did not examine the reasons for the delay in linkage to care, future research should explore this. As patients diagnosed in outpatient departments were not included in our process, there is a need to include these patients in similar types of interventions. In future, researchers should also evaluate the impact of low-cost interactions to reduce ILTFU among TB patients, especially those diagnosed in hospital, and across multiple settings.
